# Factors Associated with Ever Undergoing Colorectal Cancer Testing Among Adults in the United States

**DOI:** 10.3390/diseases14090350

**Published:** 2026-09-21

**Authors:** Kingsley Kalu, Elizabeth Ayangunna, Bushra Shah, Gulzar Shah

**Affiliations:** Department of Health Policy and Community Health, Jiann-Ping Hsu College of Public Health, Georgia Southern University, Statesboro, GA 30458, USA

**Keywords:** colorectal cancer, health disparities, cancer testing, sociodemographic characteristics, National Health Interview Survey, fecal immunochemical test, colonoscopy

## Abstract

Background: There is a growing incidence of colorectal cancer and related deaths in the United States, despite screening recommendations. This study analyzed the factors associated with having ever undergone endoscopic colorectal cancer testing among adults aged 45–85 years. Methods: This study analyzed data from the 2023 National Health Interview Survey (N = 17,087 participants). A multivariable logistic regression analysis was conducted to assess the relationship between sociodemographic and healthcare-related factors and the likelihood of having ever (a) undergone a colonoscopy or sigmoidoscopy and (b) taken a fecal immunochemical test (FIT). Results: The majority of the respondents had undergone an endoscopic colorectal exam (66%) vs. FIT (22%) in their lifetime. Compared to respondents aged 45–49 years, those in older age groups (50–85 years) were more likely to have ever undergone an endoscopic colorectal exam or FIT testing. African Americans were more likely to have ever undergone an endoscopic colorectal exam (Adjusted Odds Ratio (AOR) = 1.26, CI = 1.07–1.48), while American Indian/American Native and others (AOR = 1.35, CI = 1.02–1.80) were more likely to have ever undergone a FIT compared to White people. While education and health insurance were associated with having ever undergone an endoscopic colorectal exam, neither was associated with FIT. Compared to married respondents, widowed, separated, or divorced respondents (AOR = 0.88, CI = 0.79–0.98), and unmarried respondents (AOR = 0.70, CI = 0.60–0.82) were less likely to have ever undergone an endoscopic colorectal exam. Conclusions: Variations in factors associated with having ever undergone endoscopic colorectal cancer testing can help to understand disparities in screening uptake better and guide the development of targeted public health interventions to improve colorectal cancer prevention and early detection.

## 1. Introduction

Colorectal cancer, characterized by an unchecked abnormal cell originating in the colon or rectum, is the third most common cancer globally [1,2]. In 2020, there were around 1.93 million new cases of colorectal cancer and approximately 0.94 million deaths related to this disease worldwide [3]. The incidence of colorectal cancer is higher in highly developed countries; however, there has been an increase in cases in low- and middle-income countries, which is attributed to westernization [2,3]. Over the last three decades in the United States, the incidence of colorectal cancer among individuals under the age of 50 increased by 50%, and it is the leading cause of death among men in this age group [4,5].

In the United States, colorectal cancer is the second leading cause of death and ranks third as the most common cause of cancer-related deaths [6,7,8]. Some risk factors for colorectal cancer include being over the median age of 50 years, having a family history of the disease, inherited mutations related to colon cancer, a history of inflammatory bowel disease, diet, a history of polyps, lifestyle choices, and environmental factors (such as cigarette smoking, alcohol consumption, obesity, saturated fats, and processed red meat) [8,9,10,11,12].

As of 2022, approximately 1.4 million individuals were living with colorectal cancer in the United States [13]. In 2023, it was projected that at least 150,000 new cases of colorectal cancer would be diagnosed annually [7,13,14]. Colorectal cancer was responsible for 8.6% of all cancer deaths and an estimated 52,900 deaths in 2025 [13]. In 2020, the economic burden of colorectal cancer reached $24.3 billion [15], with medical service costs amounting to $23.7 billion and ranking second among the treatment costs for any cancer [15].

The primary methods for colorectal cancer detection are an endoscopic colorectal exam and the fecal immunochemical test (FIT) [2,7,8,16]. Colonoscopy is considered the gold standard and involves inserting a flexible scope into the rectum to examine the entire colon [8]. However, it requires significant preparation, including a special diet and bowel cleansing, and often necessitates a day off work due to sedation and recovery [2,7]. On the other hand, FIT is a non-invasive alternative that detects occult blood in stool and is easier to use, as it requires no bowel preparation or sedation, making it more convenient and generally more cost-effective [2]. While FIT is effective, it carries the risk of false-positive and false-negative results, which can lead to unnecessary anxiety and, if there is a positive FIT, further testing, including an endoscopic colorectal exam [2,7]. Evidence suggests that FIT and endoscopic colorectal exams, such as colonoscopy, were associated with lower chances of being diagnosed with or dying from colorectal cancer [7,17].

Despite the recommendations from the United States Preventive Services Task Force (USPSTF) for colorectal cancer prevention and early detection, numerous studies indicate a notable decrease in overall colorectal cancer testing and an increase in advanced-stage colorectal cancer cases [18,19,20]. This trend raises concerns and underscores the need for further research on its causes and potential prevention strategies.

Numerous studies have demonstrated a significant association between regional, racial, and ethnic disparities and colorectal cancer testing [21,22,23]. Additionally, other research examined the relationship between these disparities and the incidence and mortality rates of colorectal cancer [24,25]. Although numerous studies have examined overall colorectal cancer testing rates, there is limited research that specifically addresses the differences in the history of colorectal cancer testing, including an endoscopic colorectal exam and FIT use across sociodemographic groups. While existing studies have highlighted disparities based on factors such as race, ethnicity, socioeconomic status, and access to care, the focus on the specific usage of an endoscopic colorectal exam, including colonoscopy and sigmoidoscopy, and FIT within these contexts is often lacking.

Using a nationally representative sample, the study aimed to examine: (1) sociodemographic and healthcare-related characteristics associated with ever having undergone an endoscopic colorectal examination among U.S. adults aged 45–85 years and (2) sociodemographic and healthcare-related characteristics associated with ever having undergone FIT among U.S. adults aged 45–85 years. Using nationally representative 2023 National Health Interview Survey (NHIS) data, this study separately examined factors associated with having ever undergone an endoscopic colorectal examination and FIT, allowing for a comparison of sociodemographic and healthcare-related factors associated with each testing modality.

## 2. Materials and Methods

### 2.1. Data Source

This study utilized data from the 2023 NHIS, a nationally representative survey of the United States population that collects information on health, health behaviors, and access to healthcare among the civilian, non-institutionalized population [26]. The NHIS is the longest-running health survey in the United States, conducted annually by the National Center for Health Statistics (NCHS), which operates under the Centers for Disease Control and Prevention (CDC). This survey collects comprehensive health information from approximately 36,000 households and is administered by the United States Census Bureau. The annual Cancer Control Supplement (CCS), sponsored by the National Cancer Institute, is administered to a selected adult in each participating household. This supplement focuses on cancer-related health behaviors, covering topics such as family history of cancer, tobacco use, diet and nutrition, sun protection, physical activity, and genetic testing [27]. Since 2000, cancer control data have been collected every five years via the CCS. Due to the COVID-19 pandemic, data collection from July 2020 to April 2021 relied mainly on telephone interviews, with in-person interviews resuming in May 2021 [28]. In 2023, both face-to-face and telephone interviews were conducted, which reflects continued reliance on a mixed-mode data collection approach [29].

### 2.2. Sampling Design

The NHIS uses a multistage, stratified probability sampling design to provide a comprehensive representation of the diverse non-institutionalized population in the United States, including Hispanic, Asian, and Black households, thereby ensuring that their distinct health needs and experiences are effectively captured. The sampling framework comprises over 300 clusters of addresses that are strategically situated within well-defined geographic units, including individual counties and metropolitan areas [28].

### 2.3. Data Collection

The 2023 National Health Interview Survey data were collected from January to December 2023 to capture a snapshot of health information across the United States population [26]. The NHIS participants include sample adults aged 18 years and older, randomly selected from each household, who were asked detailed health-related questions. Each year, approximately 27,000 interviews with adults and 9000 children are expected to be completed [28]. For additional information about the questionnaire, data collection instruments, NHIS data resources, release date, validation, and application, please visit this link: https://ftp.cdc.gov/pub/Health_Statistics/NCHS/Dataset_Documentation/NHIS/2023/srvydesc-508.pdf (accessed on 27 May 2026) [28].

### 2.4. Dependent Variables

The dependent variable “Ever having undergone endoscopic colorectal exam” was operationalized using the survey question: Preventive screening: “These next questions are about colorectal cancer screening. Colonoscopy and sigmoidoscopy are exams used to detect colon cancer. Have you ever had either of these exams? “Ever had a colonoscopy or sigmoidoscopy?” The original responses were dichotomous and are coded as Yes and No.

The dependent variable “Ever having undergone Fecal Immunochemical Test” was operationalized using the survey question: “Preventive screening: The following questions are about the blood test or fecal occult blood test, fecal immunochemical, or FIT test. These are tests to determine whether you have blood in your stool or bowel movement, and can be done at home using a kit. You use a stick or brush to obtain a small amount of stool at home and send it back to the doctor or lab. Have you ever had a blood stool or FIT test using a Home test kit? Ever had a home blood stool test or FIT?” The original responses were dichotomous and are coded as ‘Yes’ and ‘No’.

### 2.5. Independent Variables

The independent variables of interest representing participants’ sociodemographic and healthcare-related characteristics are age, sex, marital status, household region, history of cancer, race, health insurance, usual source of care, family income-to-poverty ratio, urban–rural status, and education.

Age of Sample Adult: The age of the sample adult was measured using the survey question ‘age of sample adult.’ The participants’ age group included in the analysis was restricted to 45–85 years based on the updated USPSTF-recommended age for routine colorectal cancer screening [19]. The USPSTF recommends routine colorectal cancer screening for adults aged 45–75 years (Grade A for ages 50–75 years and Grade B for ages 45–49 years). For adults aged 75–85 years, the USPSTF recommends that selective screening be based on prior screening history, overall health, and preferences [19]. The 45–85-year-old age group was selected to capture self-reported history of colorectal cancer testing and examine differences in lifetime testing history across the full age range USPSTF provides screening guidance rather than adherence to guideline-recommended screening. The continuous variable was recoded into 45–49 years, 50–54 years, 55–59 years, 60–64 years, 65–69 years, 70–74 years, and 75–85 years. Participants younger than 45 years or older than 85 years were excluded from the analytic sample.

Sex: The sex of the sample adult was measured using the survey question ‘Sex of sample adult,’ which was categorized as Male or Female.

Marital status: The marital status of the sample adult was measured using the survey question ‘Legal marital status of the sample adult.’ The original response category was recoded and consolidated into four categories. Participants who reported being divorced, widowed, or separated were combined into a single category. The new response categories are married, widowed/divorced/separated, never married, and living with a partner.

Education level: Educational level was measured using the survey question ‘education’. The original educational categories were recoded into four groups: high school graduates or less, some college or an associate degree, a bachelor’s degree, and a master’s/professional/doctorate. The participants with less than a high school diploma and high school graduates/GED recipients were grouped into the “high school graduate or less” category.

Race: Race was measured using the survey question ‘race’. The original variables were White only, Black/African American only, Asian, AIAN only, AIAN and any other groups. Participants who identified as American Indian/Alaska Native (AI/AN) and other smaller racial groups were combined into the American Indian and Alaska Natives (AIAN) and others category to address small sample sizes and improve the stability and precision of multivariable regression estimates. Participants were then categorized into five racial groups: White, African American, Asian, American Indian and Alaska Natives (AIAN), and others.

History of Cancer: The history of cancer was measured using the survey question ‘Ever been told you had cancer’: Have you EVER been told by a doctor or other health professional that you had. Cancer or a malignancy of any kind? Ever been told you had cancer?” The original response options were Yes or No. The participants who reported any previous cancer diagnosis were categorized as “Yes,” while those without a reported cancer diagnosis were categorized as “No.”

Household region: The household region was measured using the survey question ‘Household region’, and responses were categorized into four standard United States Census regions, as described by the NHIS: the Northeast, Midwest, South, and West.

Health insurance: Health insurance was measured using the survey question “Are you covered by any kind of health insurance or some other kind of health care plan?” and the response categories were Yes, which indicates participants are insured, or No, which indicates participants were uninsured.

Family Income-to-poverty ratio: The family income-to-poverty ratio was measured using the survey question “Ratio of family income to poverty threshold for Sample Adult’s family.” The original response options were based on the ratio of family income to the federal poverty threshold. The responses were recoded and categorized into five income groups to reflect the socioeconomic gradients associated with healthcare access and utilization of colorectal cancer testing: Below Poverty (0.00–0.99), Near Poverty (1.00–1.99), Middle Income (2.00–3.99), High Income/Wealthy (4.00–4.99), and Highest Income (≥5.00).

Usual Place of Care: The participants’ usual place of care was measured using the survey question “What kind of place is it/do you go to most often?” and the original responses were recoded into categories: a doctor’s office or health center, an urgent care center; a clinic in a drug store or grocery store; a hospital emergency room; a VA Medical Center or VA outpatient clinic; some other place, and does not go to one place most often. Participants who most often reported not going to one place were classified as having no usual source of care.

Urban–Rural: The Urban–Rural classification was measured using the survey question “Urban-Rural Classification Scheme for Counties.” The participants’ responses were categorized into four geographical classifications: large central metropolitan, large fringe metropolitan, medium and small metropolitan, and non-metropolitan.

### 2.6. Statistical Methods/Analysis

Descriptive analysis and multivariable logistic regression were conducted to analyze the data. Descriptive statistics such as weighted percentages and unweighted frequencies were calculated to describe the study population, and multivariable logistic regression was conducted to examine the association between sociodemographic and healthcare-related characteristics and having ever undergone colorectal cancer testing (endoscopic colorectal exam and FIT).

Although there were 27,000 adult interviews, the analysis was restricted to the adults aged 45 to 85 years subpopulation who responded to questions about endoscopic colorectal testing and had no missing observations (N = 17,087). After restriction to the subpopulation of interest, the primary analytic sample included 17,087 respondents; however, the usual-place-of-care variable was available for 16,211 respondents, with 876 observations (5.13%) missing.

Given the complex sampling design of the NHIS, sampling weights that accounted for the strata and sampling units provided by the dataset owners were applied during data analysis to create accurate point estimates and ensure that the results represent the total population of the United States. The STATA svyset command, which includes the weight, strata, and primary sampling units, was included in the analysis to account for the complex sampling design of the survey. The data analysis was conducted using Stata/SE version 19.0 (Stata Corp LLC, College Station, TX, USA), with a significance threshold of *p* ≤ 0.05.

The survey-adjusted goodness-of-fit results for both logistic regression models showed high *p*-values (*p* > 0.05), suggesting that the models fit the data well and that there is no statistically significant difference between the observed and expected values. In addition, no multicollinearity was detected in either regression model.

## 3. Results: Descriptive Statistics and Respondent Characteristics

**Figure 1:**
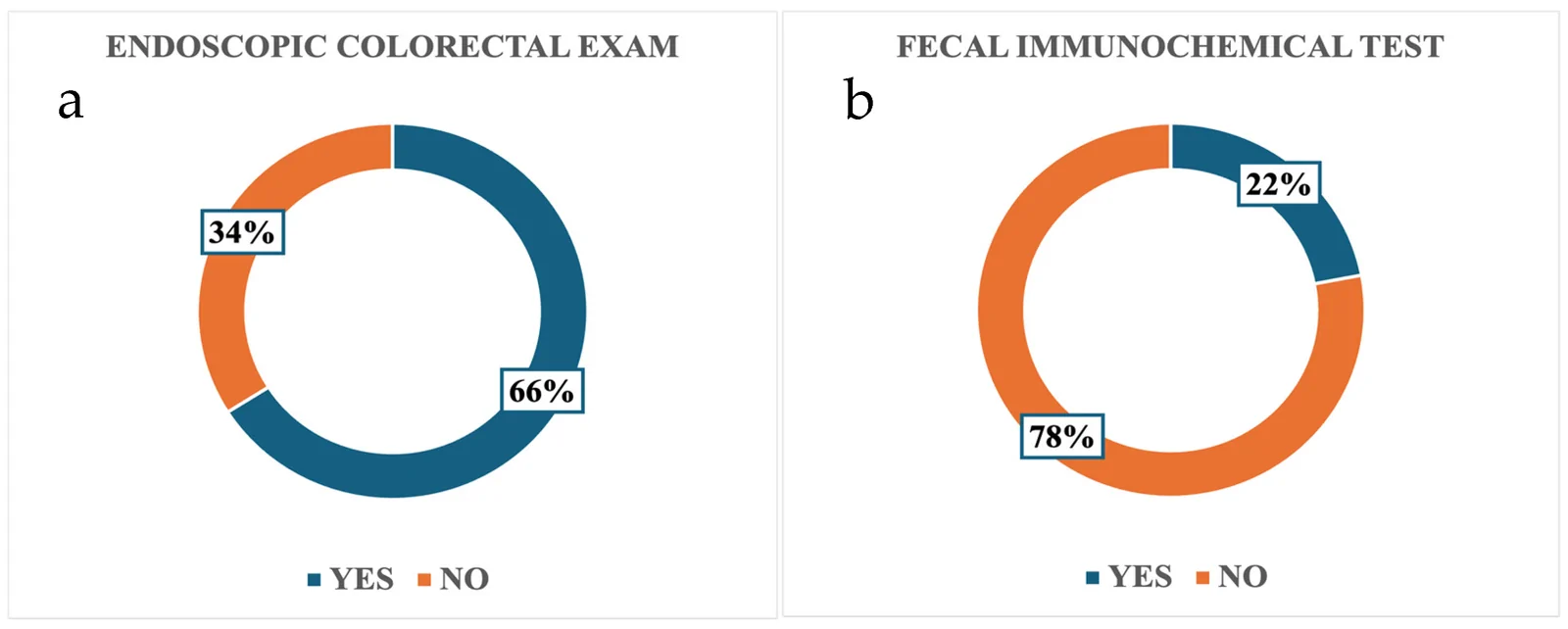
(**a**,**b**) Percent of respondents who are adults (N = 17,087) who ever had an endoscopic colorectal exam (n = 11,905) and the FIT (n = 4189).

The results in Figure 1a show that sixty-six percent (66%) of the respondents had ever done an endoscopic colorectal exam (n = 11,905), and Figure 1b shows that twenty-two percent (22%) of the respondents had ever done a FIT (n = 4189). Seventy-three percent (73%) of the respondents had done either an endoscopic colorectal exam or a FIT (n = 13,010), sixteen percent (16%) of the respondents had a history of having undergone an endoscopic colorectal exam and FIT (n = 3084), and twenty-seven percent (27%) of the respondents had neither undergone an endoscopic colorectal exam nor FIT (n = 4077).

Table 1 indicates that the majority of the adult respondents were female (52%). In terms of marital status, sixty-two percent (62%) of the respondents were married, and twenty-five percent (25%) were widowed/separated/divorced. Regarding education levels, thirty-seven percent (37%) of the respondents had at least a high school diploma, twenty-nine percent (29%) held a college degree, twenty percent (20%) possessed a bachelor’s degree, and fourteen percent (14%) had a master’s or professional degree. Thirty-nine percent (39%) resided in the Southern region, twenty-two percent (22%) in the Midwest region, followed by the Western region (21%), and eighteen percent (18%) in the Northeast region. Seventeen percent (17%) of the respondents were 75–85 years, sixteen percent (16%) were aged 60–64 years, and an equal proportion of fourteen percent (14%) were aged 50–54 years, 55–59 years, and 65–69 years. The majority of the respondents identified as white (80%), followed by African Americans (11%). Thirty-six percent (36%) of respondents’ family income-to-poverty ratio was in the highest income category, and twenty-nine percent (29%) had a family income-to-poverty ratio in the middle-income category. Seventeen percent (17%) had a near-poverty family income-to-poverty ratio. Ninety-six percent of the respondents had health insurance, and ninety percent (90%) had utilized the doctor’s office. Thirty-one percent (31%) of the respondents reside in medium and small metropolitan areas, twenty-seven percent (27%) in large central metropolitan areas, and twenty-six percent (26%) in large fringe metropolitan areas.

Table 2 shows that, compared to married respondents, widowed/separated/divorced (Adjusted Odds Ratio (AOR) = 0.88, CI = 0.79–0.98) and unmarried respondents (AOR = 0.70, CI = 0.60–0.82) were less likely to have ever had an endoscopic colorectal exam. Compared to respondents with a high school degree or less, the odds were higher for respondents with some college or an associate degree (AOR = 1.31, CI = 1.17–1.47), bachelor’s degree (AOR = 1.50, CI = 1.32–1.70), and master’s or professional degrees (AOR = 1.85, CI = 1.59–2.15) to have ever had an endoscopic colorectal exam done. Compared to respondents who identified as White, African Americans (AOR = 1.26, CI = 1.07–1.48) were more likely to have ever had an endoscopic colorectal exam.

However, respondents who identified as Asian (AOR = 0.50, CI = 0.41–0.61) were less likely to have ever had an endoscopic colorectal exam done. Compared to respondents with no history of cancer, the odds of having ever had an endoscopic colorectal exam done were higher for respondents with a history of cancer (AOR = 1.75, CI = 1.54–1.99). Compared with residents of the Western region, those residing in the Northeast (AOR = 1.27, CI = 1.10–1.47) and Midwest (AOR = 1.19, CI = 1.03–1.37) regions were more likely to have ever had an endoscopic colorectal exam. Compared to respondents aged 45–49 years, odds were higher for respondents aged 50–54 years (AOR = 2.08, CI = 1.77–2.44), 55–59 years (AOR = 4.35, CI = 3.72–5.09), and 60–64 years (AOR = 6.50, CI = 5.50–7.68), 65–69 years (AOR = 8.58, CI = 7.22–10.19), 70–74 years (AOR = 10.24, CI = 8.56–12.25) and 75–85 years (AOR = 9.08, CI = 7.68–10.73) to have ever had an endoscopic colorectal exam done. Compared to the respondents whose family income was below the poverty level, those whose family income was near poverty (AOR = 1.29, CI = 1.07–1.55), middle income (AOR = 1.43, CI = 1.20–1.71), high income (AOR = 1.42, CI = 1.15–1.74), and highest income (AOR = 1.98, CI = 1.63–2.39) were more likely to have ever had an endoscopic colorectal exam done. Compared with respondents without healthcare coverage, those with healthcare coverage (AOR = 2.72, CI = 2.06–3.59) were more likely to have ever had an endoscopic colorectal exam. Compared with respondents who utilized a doctor’s office/health center, respondents who utilized urgent care in a drug/grocery store (AOR = 0.66, CI = 0.55–0.80) and those who utilized other healthcare services or did not go to any healthcare service (AOR = 0.58, CI = 0.34–0.98) had lower odds of having ever had an endoscopic colorectal exam.

Compared to respondents with no history of cancer, the odds of having ever had a FIT done were higher for respondents with a history of cancer (AOR = 1.24, CI = 1.12–1.38). Compared with respondents in the Western region, those residing in the Northeast region (AOR = 0.47, CI = 0.40–0.55), the Midwest region (AOR = 0.46, CI = 0.40–0.54), and the South region (AOR = 0.55, CI = 0.48–0.63) were less likely to have ever had a FIT. Compared to respondents aged 45–49 years, odds were higher for study participants aged 50–54 years (AOR = 1.37, CI = 1.10–1.72), 55–59 years (AOR = 1.77, CI = 1.43–2.19), 60–64 years (AOR = 2.02, CI = 1.62–2.51), 65–69 years (AOR = 2.91, CI = 2.37–3.57), 70–74 years (AOR = 3.07, CI = 2.51–3.76) and 75–85 years (AOR = 2.63, CI = 2.17–3.20) of having ever had a FIT done. Compared with respondents who identified as White, American Indians and Alaska Natives and others (AOR = 1.35, CI = 1.02–1.80) were more likely to have ever had a FIT done. Those whose family income-to-poverty ratio was near the poverty level had higher odds (AOR = 1.21, CI = 1.01–1.44) of ever having a FIT done (vs. those with a family income-to-poverty ratio below the poverty level). Compared with respondents whose usual place of care was a doctor’s office/health center, those who used urgent care at a drug/grocery store (AOR = 0.78, CI = 0.63–0.97) had lower odds of having ever had a FIT. However, those who used a VA medical center/outpatient clinic (AOR = 1.82, CI = 1.40–2.36) had higher odds of having ever had a FIT performed. Odds of FIT were lower in respondents who reside in medium and small metropolitan areas (AOR = 0.74, CI = 0.65–0.85) and non-metropolitan areas (AOR = 0.63, CI = 0.54–0.74) rather than large central metropolitan areas.

## 4. Discussion

This study assessed the sociodemographic and healthcare-related characteristics associated with having ever undergone an endoscopic colorectal exam and FIT. More respondents had ever had an endoscopic colorectal exam (66%) than a FIT (22%). The results showed that age, race, family income, usual place of care, history of cancer, and household region were significantly associated with the odds of having ever had an endoscopic colorectal exam or FIT done. Previous studies from the United States indicated that colorectal cancer testing rates increased among different racial and ethnic groups, as well as in the age group 45–49 years, from 2019 to 2023 [30]. Our findings showed that the 50 to 74 years age group had increasingly higher odds of having ever had an endoscopic colorectal exam or FIT testing, after which it slightly dropped for the 75–85 years age group. These findings likely reflect cumulative exposure opportunity over time rather than true differences in ever undergoing any tests across age groups. The authors found that respondents in the 70–74 age group were most likely to have ever had an endoscopic colorectal exam or FIT. A healthcare provider recommendation or presenting with symptoms suggestive of colorectal cancer may have led to this finding [31,32,33]. It is estimated that people aged 65 years and older have about a threefold increased risk of developing colorectal cancer compared to those aged 50–64 years, and a thirtyfold greater risk when compared to people aged 25–49 years, highlighting the importance of continued adherence to recommended screening guidelines across eligible age groups [34]. Older individuals are likely to have had more testing opportunities as they grow older, e.g., following a doctor’s orders for preventive or diagnostic purposes. Early detection will reduce cancer mortality and should be promoted across the high-risk age groups.

The findings support previous studies that adults in the United States with higher educational levels were more likely to have ever undergone an endoscopic colorectal exam [35,36]. Higher levels of health literacy may have contributed to respondents undergoing an endoscopic colorectal exam due to its accuracy, physician endorsements, direct visualization of precancerous lesions, and the ability to remove abnormal tissues, such as polyps, during the procedure [37]. Public health practitioners should explore FIT testing programs, as they are non-invasive, low-cost, and likely more acceptable to the general population.

The results aligned with previous studies that found that respondents who were widowed, separated, divorced, or never married were less likely to have ever undergone an endoscopic colorectal exam. This could be due to various factors, including limited social support, financial constraints, and an increased likelihood of facing other health issues [38,39]. The invasive nature of an endoscopic colorectal exam requires a minor hospital procedure/anesthesia, and individuals with minimal to no social support needed for the procedure could consider a FIT. Furthermore, this may be partly explained by the younger age group among never-married participants, which could contribute to their lower likelihood of having ever undergone an endoscopic colorectal exam. This study showed significant racial disparities in history of having undergone colorectal cancer testing, including an endoscopic colorectal exam or FIT: African American respondents were more likely to have ever had an endoscopic colorectal exam. In contrast, Asian respondents were less likely to opt for this method. Additionally, respondents in the combined AIAN and other racial groups category had higher odds of having ever undergone FIT. Given the heterogeneity of this combined category, this finding should be interpreted with caution. There is mixed evidence regarding the racial disparities in colorectal cancer testing, with some showing lower testing rates among racial minorities and a few showing higher testing rates among African Americans in select population groups, e.g., veterans [40,41]. African Americans being more likely to have ever undergone an endoscopic colorectal exam could be due to the high incidence and mortality rate of colorectal cancer among this group and subsequent increased awareness of testing methods [24]. Among Asian-Americans, colorectal cancer is the second most frequently diagnosed cancer and a leading cause of cancer-related death; the relative lack of colorectal cancer testing is a cause for concern [42,43]. These variations highlight the importance of understanding the diverse needs of different racial and ethnic groups and how they can affect the accessibility and timeliness of colorectal cancer testing.

Our findings align with previous research that respondents with a personal history of cancer were more likely to have ever undergone colorectal cancer testing, including an endoscopic colorectal exam and FIT [36]. In addition, our study demonstrated that respondents had nearly a twofold higher likelihood of having ever had an endoscopic colorectal exam. While this does not necessarily indicate greater preventive awareness or adherence to recommended colorectal cancer testing, individuals with a history of cancer may have more frequent healthcare encounters, follow-up care, or physician recommendations which may increase opportunities for colorectal cancer testing.

The authors found that respondents living in the Northeast, South, and Midwest were less likely to have ever undergone a FIT, which is similar to a study conducted among United States veterans [23]. However, our findings also revealed that respondents living in the Northeast and Midwest regions were more likely to have ever undergone an endoscopic colorectal exam. Regional variations in the history of colorectal cancer testing raise important questions about the factors that influence these decisions, including regional healthcare access, physician recommendations, and patient awareness of testing options. Understanding these dynamics is vital to improving uptake of recommended colorectal cancer testing and ensuring eligible individuals receive recommended preventive care.

The study is consistent with previous findings that showed an association between income/poverty levels and colorectal cancer testing [44]. The family income-to-poverty ratio, a proxy for household income, was increasingly associated with a higher likelihood of having ever undergone an endoscopic colorectal exam. Recommendations for colorectal cancer testing or diagnostic evaluation from a healthcare provider could explain the association, regardless of the family income-to-poverty ratio. However, only respondents with a near-poverty family income-to-poverty ratio were more likely to have ever had a FIT. This may indicate the accessibility and relative ease of doing a FIT.

Having health insurance was associated with having ever undergone only an endoscopic colorectal exam and not a FIT. The relatively higher cost of care associated with an invasive procedure, such as a colonoscopy/sigmoidoscopy, necessitates health insurance and supports past research that found health insurance was associated with colorectal cancer testing [44]. Respondents whose usual source of care was urgent care at a drug or grocery store were less likely to have ever had an endoscopic colorectal exam or FIT, and those who received care elsewhere or did not go to one place were less likely to have ever had an endoscopic colorectal exam. A study found [45] that having a primary care physician and a usual source of care were associated with colorectal cancer testing. Policies and programs that promote healthcare access could reduce the cancer incidence rate. Respondents whose usual source of care was a VA medical center or outpatient clinic were more likely to have ever had a FIT. This finding may be attributable to a FIT mailing program for colorectal cancer testing across several VA hospitals [46,47,48,49]. Scaling this FIT mailing program to include non-VA hospital systems could improve colon cancer testing.

The findings showed that medium & small metropolitan and non-metropolitan areas were less likely to have ever had a FIT. This is consistent with a previous study [50] that found individuals in rural areas were less likely to have undergone any colorectal cancer testing. Disparities in colorectal testing among rural dwellers can be reduced by expanding access to tests such as FIT.

Our study findings provide practice- and policy-relevant information, specifically for the design of colorectal cancer testing programs. The results suggest that public health practitioners and health systems should prioritize targeted outreach strategies. These strategies must focus on addressing population groups with a lower history of colorectal cancer testing, particularly individuals with limited social support, lower educational attainment, racial minorities, or those residing in regions with historically lower testing participation. Expanding awareness of available colorectal cancer testing options may help increase participation. In particular, promoting less invasive options, such as the fecal immunochemical test (FIT), may encourage testing among individuals who face barriers to cost, procedural burden, or access to care. In addition, community-based education, provider recommendation, and culturally tailored communication strategies remain critical tools for improving colorectal cancer testing.

Despite the strengths of the study, the findings should be interpreted in consideration of several limitations. The study used cross-sectional secondary data to assess lifetime exposure to these tests, and the other variables may have been measured after the tests, so it cannot establish causation or provide longitudinal information on colorectal cancer testing adherence. Self-reported data could have introduced recall bias, and the observed age differences may partly reflect exposure-time bias. The NHIS dataset does not include whether an endoscopic colorectal exam or FIT was performed for routine, diagnostic evaluation, or surveillance, and our findings should be interpreted as factors associated with having undergone colorectal cancer testing experience rather than guideline-concordant testing behavior. The NHIS history-of-cancer variable does not identify cancer type; therefore, respondents with a history of colorectal cancer could not be separately identified, which may have influenced the observed association between cancer history and colorectal cancer testing. Analysis was limited to variables available in the secondary dataset, and potentially important factors, including family history of colorectal cancer, symptoms prompting testing, comorbidity burden, and provider recommendation, could not be assessed. An endoscopic colorectal exam and FIT are often considered alternative colorectal cancer testing modalities. More qualitative information is needed as to why respondents may prefer one testing method over another. Another limitation is the selective use of colorectal cancer testing among adults aged 76–85 years based on patient preferences and clinical judgment, while routine colorectal cancer testing for adults aged 45–75 years may introduce variability in testing estimates among older adults. Thus, the observed testing patterns should not be interpreted as differences in adherence to routine screening recommendations. Although the NHIS dataset is nationally representative, subgroup estimates, especially racial subgroups with small sample sizes, had to be combined, and findings should be interpreted with caution as differences in colorectal cancer testing patterns among these populations may be obscured.

## 5. Conclusions

Colorectal cancer continues to be a significant and preventable cause of morbidity and mortality in the United States. In this study, key sociodemographic and healthcare-related characteristics associated with having ever undergone colorectal cancer tests included marital status, educational attainment, race, and geographic region. Differences were also observed in the use of specific colorectal cancer testing modalities, suggesting that access, awareness, and social support may influence individuals’ engagement with recommended testing options. Although the study was not based on testing frequency that adhered to the USPSTF, the findings suggest that increasing the uptake of colorectal cancer testing must remain a key priority in population health. Addressing these disparities requires coordinated strategies that address social and cultural inequities, rather than focusing solely on individual behavior modification to improve colorectal cancer testing uptake. The study findings highlight populations such as racial minorities and rural residents that may benefit from targeted, equity-focused public health and health system approaches that address potential barriers and support timely access to colorectal cancer testing. Sustained attention to these disparities will be important for reducing the burden of colorectal cancer in the United States.

## Figures and Tables

**Table 1:** Descriptive Statistics of the Respondents.

Variables	Frequency (Unweighted)	Percentages (Weighted %)
Dependent Variable		
Colorectal exam		
History of Endoscopic colorectal exam or FIT	13,010	73
History of Endoscopic colorectal exam and FIT	3084	16
Endoscopic Colorectal Exam		
No	5182	34
Yes	11,905	66
FIT		
No	12,898	78
Yes	4189	22
Sex		
Male	7708	48
Female	9379	52
Marital Status		
Married	8415	62
Widowed/Divorced/Separated	6250	25
Never Married	1841	8
Living with a Partner	581	5
Education Level		
High school graduate/less	5860	37
Some college/associate degree	4866	29
Bachelor’s degree	3690	20
Masters/Professional degree	2671	14
Race		
White	14,040	80
African American	1904	11
Asian	754	6
AIAN and others	389	3
History of Cancer		
Yes	3300	17
No	13,787	83
Household region		
Northeast region	2667	18
Midwest region	3983	22
South region	6451	39
West region	3986	21
Age of Sample Adult		
45–49 years	1689	13
50–54 years	1882	14
55–59 years	2132	14
60–64 years	2461	16
65–69 years	2671	14
70–74 years	2435	12
75–85 years	3817	17
Rurality		
Large Central Metropolitan	4455	27
Large Fringe Metropolitan	4050	26
Medium and small metropolitan	5536	31
Nonmetropolitan	3046	16
Health Insurance Coverage		
Yes	16,496	96
No	591	4
Usual Place of Care		
Doctor’s Office/health center	14,671	90
Urgent care in a drug/grocery store	797	5
Hospital emergency room	246	2
VA medical center/outpatient clinic	381	2
Some other place/does not go to one place	116	1
Family income to poverty ratio		
Below Poverty	1572	8
Near Poverty	3097	17
Middle Income	5052	29
High income/wealthy	1773	10
Highest income	5593	36

Notes: Values are presented as frequencies (unweighted) and percentages (%). Percentages may not sum to 100 due to rounding. Abbreviations: AIAN = American Indian/Alaska Native; FIT = fecal immunochemical test. The analytic sample includes respondents with complete data for all variables included in the descriptive analysis.

**Table 2:** Multivariable Logistic Regression of Factors associated with Having Ever Undergone Endoscopic Colorectal Exam and FIT.

	Having Ever Undergone an Endoscopic Colorectal Exam				Having Ever Undergone FIT			
Independent Variables		95%CI				95%CI		
	AOR	LL	UL	*p*-value	AOR	LL	UL	*p*-value
Sex of Sample Adult								
Male	Ref. Category				Ref. Category			
Female	1.01	0.92	1.11	0.90	1.00	0.91	1.09	0.96
Education Level of the Sample Adult								
High school graduate or less	Ref. Category				Ref. Category			
Some college or associate degree	**1.31**	1.17	1.47	<0.01	1.08	0.97	1.22	0.16
Bachelor’s degree	**1.50**	1.32	1.70	<0.01	1.10	0.96	1.26	0.16
Master’s or Professional degree	**1.85**	1.59	2.15	<0.01	1.13	0.98	1.30	0.08
Age of Sample Adult								
45–49 years	Ref. Category				Ref. Category			
50–54 years	**2.08**	1.77	2.44	<0.01	**1.37**	1.10	1.72	0.01
55–59 years	**4.35**	3.72	5.09	<0.01	**1.77**	1.43	2.19	<0.01
60–64 years	**6.50**	5.50	7.68	<0.01	**2.02**	1.62	2.51	<0.01
65–69 years	**8.58**	7.22	10.19	<0.01	**2.91**	2.37	3.57	<0.01
70–74 years	**10.24**	8.56	12.25	<0.01	**3.07**	2.51	3.76	<0.01
75–85 years	**9.08**	7.68	10.73	<0.01	**2.63**	2.17	3.20	<0.01
Marital Status								
Married	Ref. Category				Ref. Category			
Widowed/Separated/Divorced	**0.88**	0.79	0.98	0.02	1.08	0.98	1.20	0.11
Never Married	**0.70**	0.60	0.82	<0.01	1.00	0.85	1.15	0.90
Living with a Partner	0.93	0.75	1.17	0.54	1.02	0.81	1.29	0.86
Race								
White	Ref. Category				Ref. Category			
African American	**1.26**	1.07	1.48	0.01	1.12	0.97	1.29	0.13
Asian	**0.50**	0.41	0.61	<0.01	1.01	0.82	1.24	0.91
AIAN and Others	0.87	0.60	1.25	0.45	**1.35**	1.02	1.80	0.04
History of Cancer								
No	Ref. Category				Ref. Category			
Yes	**1.75**	1.53	1.99	<0.01	**1.24**	1.12	1.38	<0.01
Household Region								
West	Ref. Category				Ref. Category			
Northeast	**1.27**	1.10	1.47	<0.01	**0.47**	0.40	0.55	<0.01
Midwest	**1.19**	1.03	1.37	0.02	**0.46**	0.40	0.54	<0.01
South	1.12	0.98	1.27	0.11	**0.55**	0.48	0.63	<0.01
Family income-to-poverty ratio								
Below Poverty	Ref. Category				Ref. Category			
Near Poverty	**1.29**	1.07	1.55	0.01	**1.21**	1.01	1.44	0.04
Middle Income	**1.43**	1.20	1.71	<0.01	1.08	0.92	1.28	0.34
High Income/Wealthy	**1.42**	1.15	1.74	<0.01	0.99	0.81	1.22	0.92
Highest Income	**1.98**	1.63	2.39	<0.01	0.85	0.72	1.02	0.08
Health Insurance Coverage								
No	Ref. Category				Ref. Category			
Yes	**2.72**	2.06	3.59	<0.01	1.41	0.99	1.99	0.05
Usual Place of Care								
Doctor’s office/health center	Ref. Category				Ref. Category			
Urgent care in a drug/grocery store	**0.66**	0.55	0.80	<0.01	**0.78**	0.63	0.97	0.03
Hospital emergency room	0.73	0.50	1.09	0.12	0.71	0.47	1.08	0.11
VA Medical Center/outpatient clinic	1.18	0.86	1.63	0.31	**1.82**	1.40	2.36	<0.01
Some other place/Does not go to one place	**0.58**	0.34	0.98	0.04	0.95	0.55	1.66	0.87
Rurality								
Large central metropolitan	Ref. Category				Ref. Category			
Large fringe metropolitan	1.04	0.91	1.19	0.56	0.91	0.79	1.04	0.16
medium and small metropolitan	1.10	0.97	1.25	0.15	**0.74**	0.65	0.85	<0.01
Nonmetropolitan	0.93	0.80	1.07	0.32	**0.63**	0.54	0.74	<0.01
Mean VIF	1.10				1.59			
Survey-adjusted Goodness of Fit	014				0.82			
The reference outcome category is No.								

Notes: Bold AORs indicate statistical significance at *p* ≤ 0.05. Survey weights were applied to account for the complex sampling design of the NHIS. Survey-adjusted goodness-of-fit indicated acceptable model fit. Abbreviations: AOR = adjusted odds ratio; CI = confidence interval; LL = lower limit; UL = upper limit; Ref. = reference category; FIT = fecal immunochemical test; AIAN = American Indian/Alaska Native.

## Data Availability

The data analyzed in this study are publicly available from the National Health Interview Survey (NHIS), conducted by the National Center for Health Statistics (NCHS) of the Centers for Disease Control and Prevention. The dataset used for this study (NHIS 2023) is available at https://www.cdc.gov/nchs/nhis/documentation/2023-nhis.html (accessed on 31 December 2025).

## Abbreviations

The following abbreviations are used in this manuscript:

CRC	Colorectal Cancer
FIT	Fecal Immunochemical Test
NHIS	National Health Interview Survey
NCHS	National Center for Health Statistics
CDC	Centers for Disease Control and Prevention
USPSTF	United States Preventive Services Task Force
AOR	Adjusted Odds Ratio
CI	Confidence Interval
AIAN	American Indian and Alaska Native
